# Comprehensive analysis of the cardiac proteome in a rat model of myocardial ischemia-reperfusion using a TMT-based quantitative proteomic strategy

**DOI:** 10.1186/s12953-020-00158-4

**Published:** 2020-03-06

**Authors:** Sun Ha Lim, Jongwon Lee, Mee-Jung Han

**Affiliations:** 1 Department of Biochemistry, School of Medicine, Catholic University of Daegu, 33, 17-gil, Duryugongwon-ro, Nam-gu, Daegu, 42472 Republic of Korea; 2 Department of Biomolecular and Chemical Engineering, Dongyang University, 145 Dongyang-daero, Punggi-eup, Yeongju, Gyeongbuk 36040 Republic of Korea; 3 Department of Nursing, Dongyang University, 145 Dongyang-daero, Punggi-eup, Yeongju, Gyeongbuk 36040 Republic of Korea

**Keywords:** Cardiac proteome, TMT labeling, Quantitative proteomics, Ischemia, Myocardial infarction, *Triticum aestivum*, Dietary fiber

## Abstract

**Background:**

Traditional studies of the cardiac proteome have mainly investigated in an animal model by two-dimensional gel electrophoresis (2-DE). However, the results have not been of satisfactory quality for an understanding of the underlying mechanism. Recent quantitative proteomic methods have been improved to overcome these limitations. To comprehensively study the cardiac proteome in a rat model of ischemia-reperfusion (IR), we developed a tandem mass tag (TMT)-based quantitative proteomic strategy. Furthermore, using this strategy, we examined the molecular mechanisms underlying the prevention of myocardial infarction by the intake of *Triticum aestivum* L. extract (TALE), a representative dietary fiber grain.

**Methods:**

Cardiac proteomes were analyzed by 2-DE as a gel-based approach, and TMT labeling coupled with two-dimensional liquid chromatography (2D-LC) and tandem mass spectrometry (MS/MS) as a non-gel-based quantitative approach. Additionally, gene ontology annotation was conducted by PANTHER database. Several proteins of interest were verified by a Western blot analysis.

**Results:**

Total 641 proteins were identified commonly from two independent MS datasets using 2D-LC MS/MS. Among these, we identified 151 IR-related proteins that were differentially expressed between the sham-operation group and IR group, comprising 62 up-regulated proteins and 89 down-regulated proteins. Most of the reduced proteins were involved in metabolic processes. In addition, 57 of the IR-related proteins were affected by TALE intake, representing 25 up-regulated proteins and 32 down-regulated proteins. In particular, TALE intake leads to a switch in metabolism to reduce the loss of high-energy phosphates and the accumulation of harmful catabolites (especially reactive oxygen species (ROS)) and to maintain cytoskeleton balance, leading to a reduction in cardiac IR injury.

**Conclusions:**

Our study provides a comprehensive proteome map of IR-related proteins and potential target proteins and identifies mechanisms implicated in the prevention of myocardial infarction by TALE intake in a rat IR model.

## Background

Cardiac ischemia and ischemia-reperfusion (IR) injury are major contributors to morbidity and mortality worldwide [1]. Ischemia results from an imbalance between myocardial oxygen demand and supply, causing cardiac dysfunction, arrhythmias, myocardial infarction, and sudden death. In fact, acute myocardial infarction is a primary cause of death worldwide [2]. Specifically, the blood supply to heart muscle cells is typically reduced by an occlusion in the coronary artery, leading to myocardial infarction. Reperfusion therapy removes the occlusion by thrombolytic therapy, bypass surgery or percutaneous coronary intervention and is currently the mainstay of treatment for acute myocardial infarction. Clinically, early diagnostic markers of ischemic heart disease and myocardial infarction and pharmacological postischemic treatments have been widely investigated to reduce infarction size and mortality after IR injury [3, 4]. However, the pathogenesis of myocardial infarction and its progression to heart failure are complex multifactorial processes that largely depend on the size of the infarcted myocardium.

The genes associated with the control of apoptotic cascades (p38, c-Jun N-terminal kinase (JNK), mitogen-activated protein (MAP) kinases [5], Bcl-2, Bax, caspase-3 and DNA nicking [6]) are closely associated with reducing myocardial infarction as demonstrated using molecular analysis tools, such as Western blotting and immunohistochemistry. Additionally, alterations in metabolic pathways and apoptotic cascades by IR injury can be more deeply analyzed at the proteome level. Most IR-related proteomic studies have utilized a 2-D gel-based method [7–10]. However, traditional two-dimensional gel electrophoresis (2-DE) analyses have limitations, including gel-to-gel variations, issues with quantification based on spot intensity, and the lack of detection of a trace amount of proteins in most cases. Moreover, only several proteins exhibiting differential expression under various conditions have been identified by mass spectrometry (MS). Therefore, obtaining a systemic understanding of the mechanism underlying myocardial IR injury is still challenging.

However, recent advancements in proteomic technologies and instrumentation have radically improved throughput and detection limits to solve these problems. One of the most popular methods for relative quantification by using MS involves isobaric chemical labeling of biological samples prior to MS analysis, such as isobaric tags for relative and absolute quantitation (iTRAQ) or tandem mass tag (TMT) [11]. Moreover, the ability to multiplex with isobaric mass tags has expanded the applicability of this method to a wide range of sample types and multiple biological samples, which can be analyzed under the same chromatography and MS conditions. A limited number of more recent studies have employed liquid chromatography (LC) MS/MS-based multidimensional protein identification. Using an iTRAQ-based quantitative proteomic approach, 22 unique proteins among 220 proteins in a sarcomere-enriched fraction differed between ischemia and nonischemia regions in rat heart tissue without reperfusion [12]. In addition, neuroproteome changes induced by the administration of a tissue plasminogen activator in a rat IR model of acute ischemic stroke have been analyzed using iTRAQ labeling [13]. However, to date, a cardiac proteomic study investigating IR injury in a rat model has not been performed using a quantitative proteomic method, particularly the TMT labeling method. Furthermore, although the intake of dietary fiber (DF) reduces the risk of coronary heart disease [14, 15] and alters the physiology of myocardial infarction in a clinically significant manner, to the best of our knowledge, no study has attempted to measure proteome changes associated with DF in IR injury.

In this study, an efficient proteomic strategy was developed for studying the cardiac proteome in a rat model of IR injury. Here, using a 2-D gel-based method or TMT labeling and MS-based quantitative proteomic method, we investigated the protein expression profiles of heart tissues in a rat model of IR injury to discover possible protein targets and the underlying mechanisms involved in the intake of *Triticum aestivum* L. extract (TALE) as a representative DF grain [16, 17]. The rats were exposed to no IR treatment (sham-operated group), IR treatment with 30 min of ischemia and 3 h of reperfusion (IR group), or TALE intake for 3 days prior to IR injury (TALE intake group) as reported in our previous studies [18, 19]. The proteomic data obtained using the TMT labeling strategy revealed more information about the metabolic pathways involved in IR, thereby increasing our understanding of the underlying mechanism by which TALE intake regulates protein expression to potentially protect the heart from serious IR injury.

## Methods

### Animals

Eight-week-old male Sprague Dawley (SD) rats were purchased from Samtaco, Inc. (Osan, Korea). The experiments were performed according to the Guidelines for the Animal Care and Use of Laboratory Animals approved by the Institutional Animal Care and Research Advisory Committee of Catholic University, Daegu, Korea (No. DCIAFCR-151230-20-Y). The animals were housed with ad libitum access to a chow diet and water under diurnal lighting conditions in a temperature-controlled environment until the start of the IR experiment.

### Myocardial infarction model

Myocardial infarction was generated by ligating the left anterior descending coronary artery (LAD) in male SD rats (~ 300 g) as previously described [18, 20, 21]. Briefly, the rats were anesthetized with intraperitoneal injections of ketamine (100 mg/kg) and xylazine (5 mg/kg). The rats were ventilated with air after intubation. The LAD was ligated at approximately 5 mm below the aortic origin. In the IR experiments, the hearts were ligated for 30 min and reperfused for 3 h. During surgery, the rectal temperature was maintained between 37 ± 0.5 °C with a thermostatically controlled warming plate (Harvard Apparatus, Holliston, MA).

### Infarct size assessments

The infarct size was assessed by 2,3,5-triphenyltetrazolium chloride (TTC) staining [18, 20, 21]. Evans blue dye was infused, and areas at risk (AAR) exhibiting no infiltration of Evans blue dye were assessed. Two slices prepared by cutting the myocardium were stained with TTC, and the infarct area (IA), which is the region not stained with TTC, was assessed. The areas of the left ventricle (LV), AAR, and IA were determined by computerized planimetry using ImageJ software (ImageJ version 1.47 V, NIH). Specifically, the infarct size (IS) and risk size (RS) were defined as a percentage of IA to AAR and AAR to LV, respectively.

### Protein extraction for proteome analysis

The frozen heart tissues (approximately 100 mg) were added to 0.5 mL of lysis buffer containing 8 M urea, 4% CHAPS, 5 mM magnesium acetate and 30 mM Tris (pH 8.5). The suspension was mechanically homogenized with metal beads (hard tissue grinding MK28-R, Bertin Technologies, Montigny-le-Bretonneux, France) using a Precellys 24 (Bertin Technologies) at 5800 rpm for 20 s and repeated thrice with a 5-min pause between the homogenization cycles to prevent overheating. The supernatant was collected by centrifugation at 20,800×*g* for 30 min at 15 °C. The protein concentrations of the supernatants were estimated using a Bradford protein assay (Bio-Rad, Hercules, CA, USA), and the protein extracts were stored in aliquots at − 80 °C until use.

### 2-DE and image analysis

The protein extracts (200 μg) were resolubilized with rehydration buffer containing 7 M urea, 2 M thiourea, 4% (w/v) CHAPS, 1% (w/v) DTT, and 1% (v/v) IPG buffer (pH 3–10 NL; GE Healthcare, Chalfont St. Giles, UK). The 2-DE experiments were performed using an IPGphor IEF system (GE Healthcare) and Protean II xi Cell (Bio-Rad) as previously described [22]. The prepared protein samples were carefully loaded onto IPG strips (18 cm, pH 3–10 NL; GE Healthcare). The loaded IPG strips were rehydrated for 12 h using an in-gel rehydration method and focused at 20 °C for 15 min at 250 V, followed by 8000 V until a total of 60 kV·h was achieved. The strips were equilibrated in equilibration buffers as previously described [22] and then placed on 12% (w/v) sodium dodecyl sulfate polyacrylamide gel electrophoresis (SDS-PAGE) gels. The protein spots were visualized using a silver staining kit (GE Healthcare), and the stained gels were scanned with a UMAX PowerLook 2100XL Scanner (UMAX Technologies, Inc., TX, USA). PDQuest 2-D Analysis Software (Bio-Rad) was used to automate the protein spot identification within the image and quantify the density of the spots based on the percentage of volume (i.e., the values were calculated using the integration of the spot optical intensity over the spot area). Each sample was analyzed at least in duplicate. Features resulting from nonprotein sources were removed, and the protein spots were normalized. Then, pairwise image comparisons were performed.

### TMT labeling and MS-based quantitative proteomics

The protein extracts were tagged for quantitative proteomics using an isobaric TMT six-plex reagent kit (No. 90064, Thermo Scientific) as previously described [19, 23]. The six-plex labeling scheme used allows up to six samples to be compared in a single MS run, thereby increasing the experimental throughput for protein quantitation. In this study, three samples, each containing 30 μg of peptides, were labeled in duplicate using TMT-126 and TMT-129 for the sham-operated group, TMT-127 and TMT-130 for the IR group, and TMT-128 and TMT-131 for the TALE intake group. Then, the six samples were mixed, speed-vacuum dried, and redissolved in 50 μL of water containing 0.1% formic acid.

The TMT-labeled samples were analyzed using a 2DLC-MS/MS system consisting of a Nano Acquity UPLC system (Waters, USA) and a linear trap quadrupole (LTQ) Orbitrap Elite mass spectrometer (Thermo Scientific, USA) equipped with a nanoelectrospray source as previously reported by Lee et al. [23] and Washburn et al. [24] with slight modification. Briefly, a strong cation exchange (SCX, 5 μm, 3 cm) column was used for the peptide fractionation by a salt gradient. The fractionated peptides were separated on a 200-mm homemade C_18_ capillary column (Aqua; particle size 3 μm, 100 μm silica tubing, orifice id of 5 μm). The MS data were acquired in five data-dependent collision induced dissociation-high energy collision dissociation (CID-HCD) dual MS/MS scans per full scan. For the protein identification and quantification, the MS/MS spectra were analyzed using the IPI and UniProt rat databases. The database was appended with the reversed sequences of all proteins for the false discovery rate (FDR) calculations. ProLucid [25] was used to identify the peptides with a precursor mass error of 25 ppm and a fragment ion mass error of 600 ppm. Trypsin was selected as the enzyme, with three potential missed cleavages. TMT modification (+ 229.1629) at the N-terminus and lysine residues by the labeling reagent and carbamidomethylation at cysteine were chosen as static modifications. Oxidation at methionine was chosen as the variable modification. CID and HCD tandem MS spectra from the same precursor ion are often combined by the software to allow for better peptide identification and quantification [26]. We used homemade software in which the reporter ions from the HCD spectrum were inserted into the CID spectrum with the same precursor ion as the previous scan. Reporter ions were extracted from small windows (± 20 ppm) around their expected m/z in the HCD spectrum. The output data files were filtered and sorted into a protein list by DTASelect [27] with two or more peptide assignments to obtain a protein identification and false positive rate less than 0.01. A quantitative analysis was conducted using Census in the IP2 pipeline (Integrated Proteomics, USA). To control the quality of the protein quantification, we labeled the same samples with two different TMT reagents. Comparing the different TMT labels of the same samples, data with more than 30% variation were excluded. The TMT ratios of the proteins were calculated using the average reporter ion intensities across all peptides assigned to a protein subgroup [28]. The measured intensity ratios of the proteins were transformed to a log_2_ scale.

### Immunoblotting

Immunoblotting was performed using protein extracts from different batches of rat heart tissues than those used for the TMT labeling experiments. Briefly, aliquots of protein extracts were thawed, and protein from each sample (20 μg) was separated on 10 or 15% SDS-polyacrylamide gels according to the molecular weight of a target protein. The proteins were transferred to Immuno-Blot PVDF membranes (Bio-Rad) using a wet electroblotter. The membranes were stained with MemCode reversible protein stain (Pierce Biotechnology, Waltham, MA, USA) and imaged to verify uniform protein loads and efficient electro transfer. The membranes were destained with Milli-Q water and blocked with nonfat dry milk. The membranes were incubated at 4 °C overnight with the following primary antibodies: anti-fatty acid-binding protein monoclonal antibody (FABP; Abcam, Cambridge, MA, USA), anti-PYGM polyclonal antibody (PYGM; Abclonal, Woburn, MA, USA), anti-adenylate kinase isoenzyme 1 polyclonal antibody (AK1; Aviva Systems Biology, San Diego, CA, USA), anti-myoglobin monoclonal antibody (MB; Abcam), anti-protein DJ-1 polyclonal antibody (DJ-1; Cell Signaling Technology, Danvers, MA, USA), anti-gelsolin monoclonal antibody (GSN; Abcam), and anti-ERK1 polyclonal antibody (ERK1; Santa Cruz Biotechnology; Dallas, TX, USA). Then, the membranes were incubated with HRP (horseradish peroxidase)-labeled secondary antibodies (Sigma-Aldrich, St. Louis, MO, USA) at room temperature for 1 h. The bound antibodies were detected by ECL immunoblotting detection reagent (GE Healthcare, UK). The images were captured using ChemiDoc XRS Gel Imager (Bio-Rad). The protein bands were analyzed by ImageJ software. ERK1 was employed as a loading control.

### Statistical analysis

The statistical analyses of a single comparison were performed using Student’s *t*-test. For the Western blot analysis, multiple comparisons were performed using one-way ANOVA, followed by Fisher’s Least Significant Difference (LSD) test using SPSS software (IBM SPSS Statistics; version 19, Armonk, NY, USA). The values are expressed as the mean ± SEM. *P* < 0.05 was considered indicative of statistical significance.

## Results and discussion

### Experimental design for the cardiac proteome analysis

To study the physiological change in the animals (particularly rats) due to myocardial infarction at the proteome level, a typical 2-DE approach has been used in many proteomic studies [7–10]. Recently, non-gel-based quantitative proteomic approaches (mainly iTRAQ) have been used to reveal more in-depth physiological phenomena and mechanisms of the cardiac proteome in an IR mouse model [11] or the neuroproteome in an IR rat model [13]. Using both methods, the most important step is harvesting tissue samples from animals impaired by IR and obtaining efficient and quantitative extraction of all proteins from the proteome despite diverse physical and chemical properties. However, the quantitative preparation of heart tissue is particularly challenging given that myocytes are composed of membranes (sarcolemma, mitochondrial membrane and sarcoplasmic reticulum) and myofilaments and contain large numbers of mitochondria [29]. Therefore, a method that completely disrupts various compartments and effectively extracts these proteins while maintaining high peptide recovery is highly desirable.

**Fig. 1:**
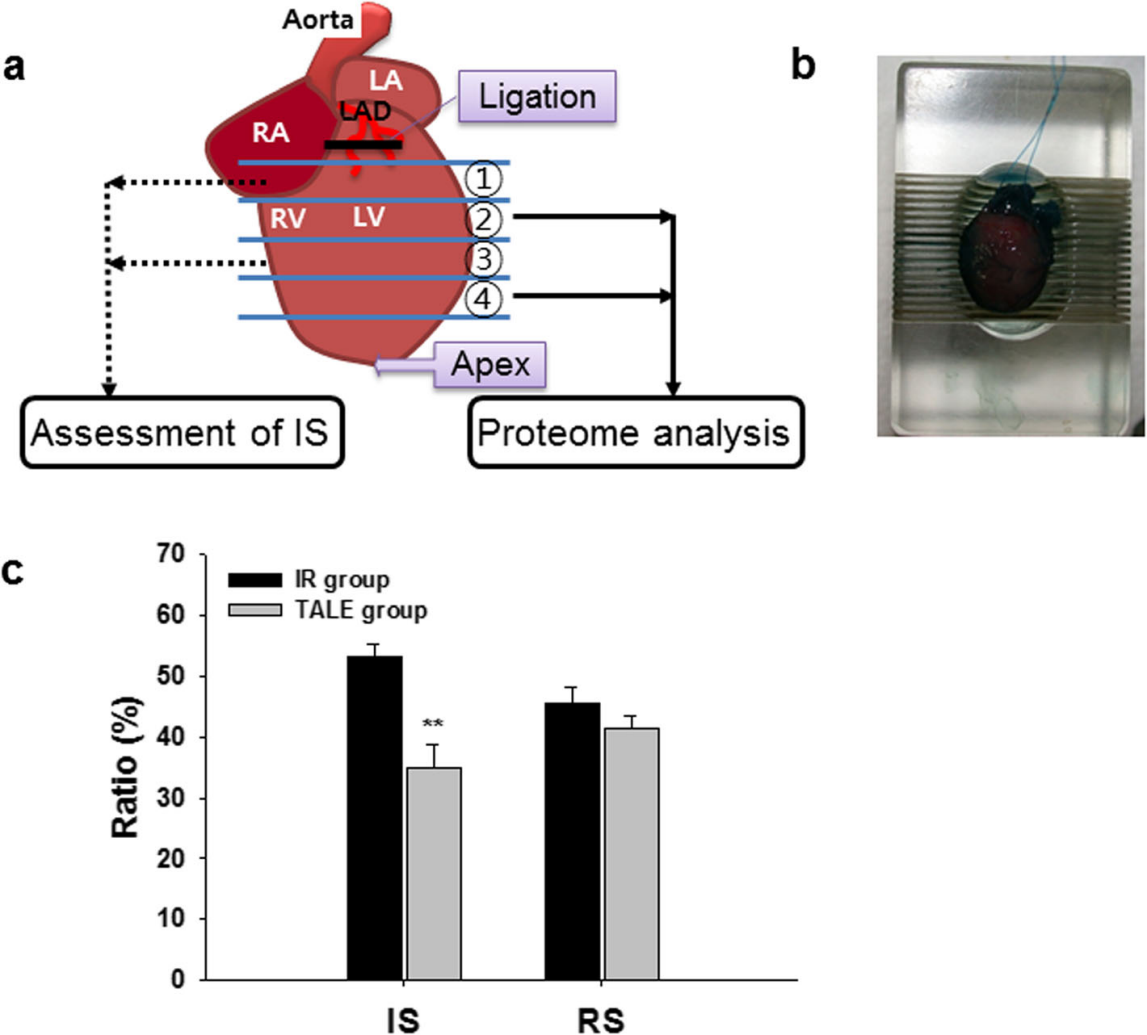
Scheme of rat heart preparation after IR experiments. **a** Rats are randomly divided into the following three groups: sham, IR, and TALE. sham, no IR but subjected to the same surgical procedure; IR, IR with surgical procedure; TALE, fed *Triticum aestivum* L. extract (TALE) as a representative DF grain prior to IR. The heart was sliced into 4 parts using a rat heart slicer matrix (ZIVIC Instruments, Pittsburgh, PA). **b** Two of the four parts were used for the assessment of the IS through TTC staining, and the remaining parts were chopped and frozen at − 80 °C. Each protein was extracted from the tissues and subjected to a proteome analysis. **c** Effect of TALE intake on the IS in an IR rat model. Abbreviations: LA, left atrium; RA, right atrium; LV, left ventricle; RV, right ventricle; LAD, left anterior descending coronary artery; IS, infart size; RS, risk size

**Fig. 2:**
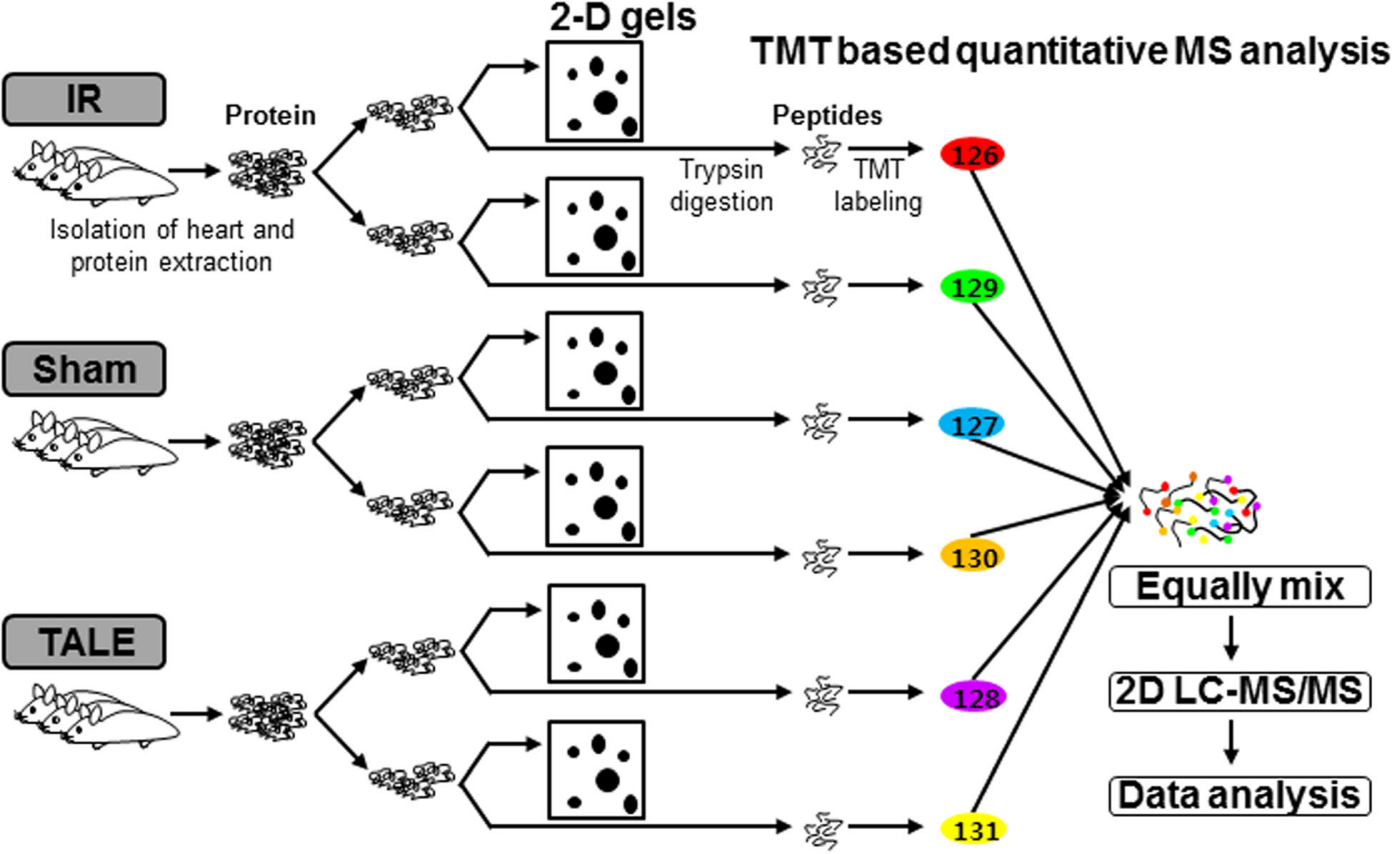
Workflow of the two proteomic approaches used in this study. Each protein was extracted from individual rats from the following groups: sham, IR, and TALE. A portion of each protein extract was subjected to 2-D gels as a part of the gel-based method. The remaining portion of each protein sample was subjected to a TMT-based quantitative strategy, such as a non-gel-based method. Equal amounts of each protein were labeled with an individual TMT and combined into one sample. The mixtures were analyzed quantitatively and qualitatively using 2DL-MS/MS. Consequently, reliable data were obtained following filtering using the statistic criteria as mentioned in the Materials and Methods

In this study, we introduce a new strategy for a more systematic proteome analysis using hard heart tissues (Fig. 1). We used an IR rat model that mimics the clinical setting in which occluded arteries are opened through reperfusion therapy, such as percutaneous coronary angioplasty [30]. In this IR model, the rats underwent ischemia (30 min) through ligation of the left anterior descending (LAD) coronary artery, followed by reperfusion (3 h) through the release of the ligation as reported in our previous studies [18, 19, 21]. The rats were randomly divided into the following 3 groups: no IR treatment (sham-operated group; *n* = 3), IR treatment with 30 min ischemia and 3 h reperfusion (IR group; *n* = 3), and TALE intake (400 mg/kg/day) for 3 days prior to IR injury (TALE intake group; *n* = 3). After performing the IR experiment, the heart was infiltrated with Evans blue dye and dissected from each rat, and then AAR assessed. The heart was sliced into 4 pieces, which were weighed (Fig. 1a and b). Two pieces were immediately used for the IS assessment with TTC. TALE intake significantly reduced the IS compared with that in the IR group (35.0 ± 3.9% versus 53.2 ± 2.0%) (Fig. 1c). The remaining two pieces were further minced with a razor blade, rapidly frozen in liquid nitrogen and stored at − 80 °C for the proteome analyses. In this proteomic study (Fig. 2), two approaches (i.e., a gel-based method and a non-gel-based quantitative method) were employed to identify a more efficient method for cardiac proteome analyses.

### Gel-based method using 2-DE

**Fig. 3:**
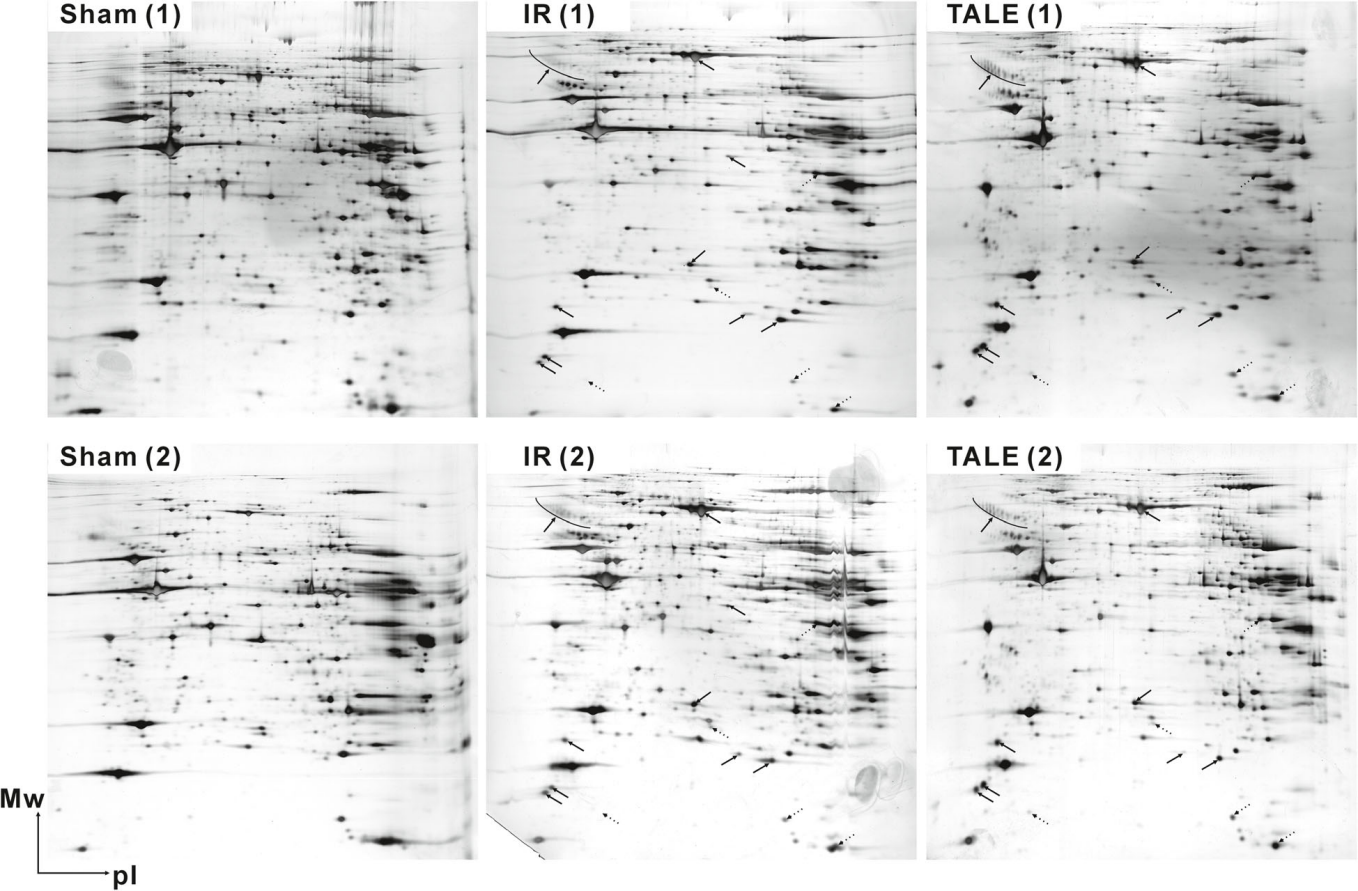
Representative 2-D gels of cardiac proteomes in an IR rat model. Repeated 2-D gels of sham (left), IR (middle), and TALE (right) groups are presented at the top/bottom of the figure. Solid and dotted arrows on the gels of the IR and TALE groups indicate increased and decreased protein levels compared with the corresponding proteins in the sham group. The positions of the molecular mass standards are indicated in kilodaltons on the vertical axes, and the approximate p*I* scales are indicated on the horizontal axes

Most proteomic studies investigating IR rat models have utilized 2-D gel and MS analyses [7–10]. Only several proteins with a relatively high abundance were identified, and these proteins were involved in heat shock stress (HSP60, peroxiredoxin-2, or protein disulfide isomerase), energy metabolism (ATP synthase components), muscle contraction (myosin components), or protein synthesis (elongation factors) [7, 8, 10]. Here, we also investigated the physiological differences in the IR experiments using 2-DE, as a scheme shown in Fig. 2. In this case, the sliced tissues were homogenized with metal beads for hard tissue grinding in buffer containing urea given its compatibility with the subsequent isoelectric focusing conditions in downstream 2-DE processes. Representative 2-D gels are presented in Fig. 3. The separation of the protein spots on the 2-D gels was good, but several spots presented with only some differences in quantity similar to results from previous studies [7, 8, 10]. Thus, these data did not facilitate an understanding of the physiological and metabolic processes at the complete level. For a more in-depth analysis, we further examined the samples using quantitative MS-based proteomics.

### Non-gel-based quantitative method using TMT labeling and MS/MS analysis

**Fig. 4:**
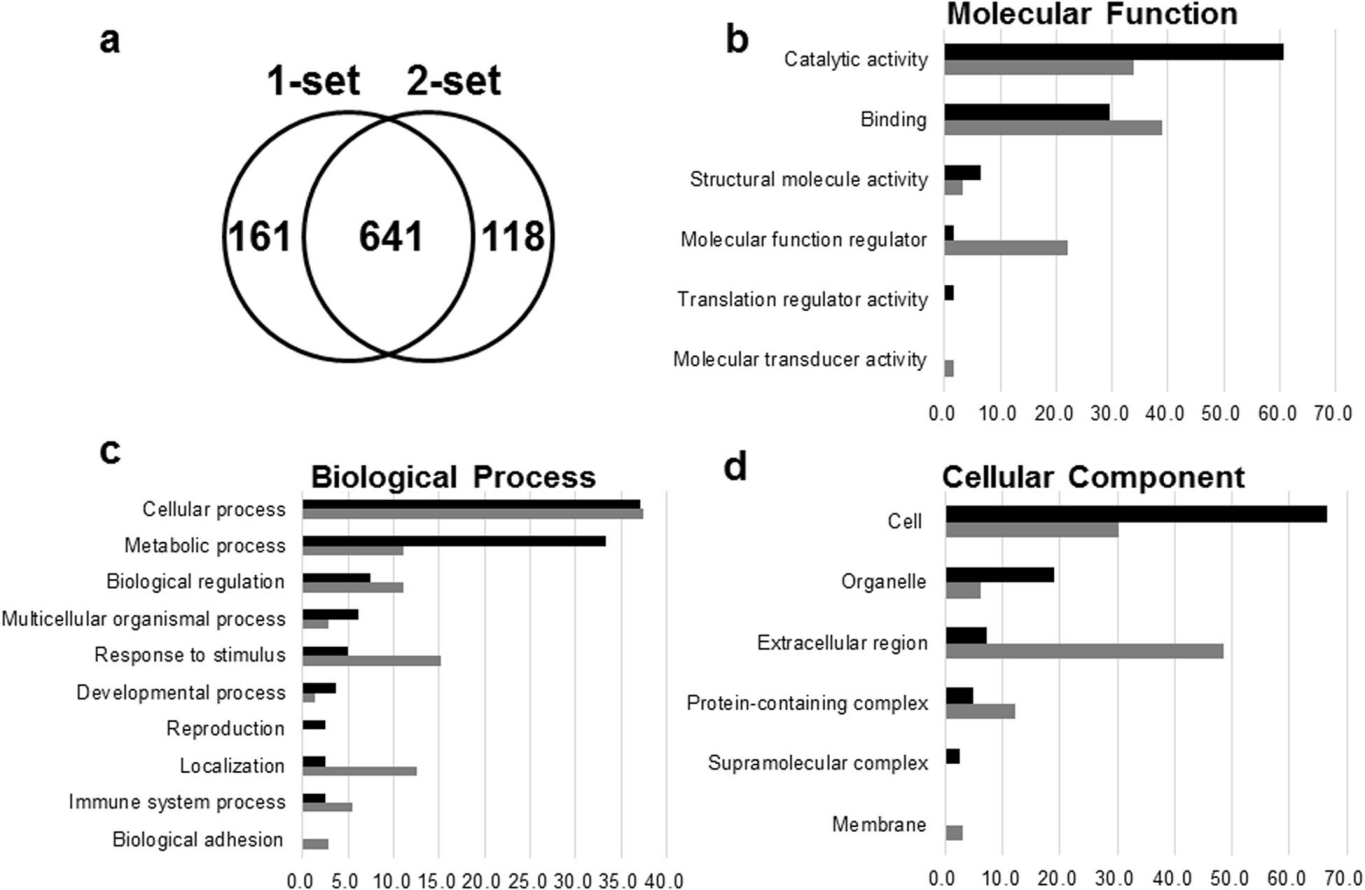
Analysis of proteins identified by a TMT-based quantitative MS. **a** Venn diagrams indicating the number of proteins identified from two different MS datasets. **b**-**d** PANTHER classification system analysis of proteins up- and down-regulated in the IR rat model: (**b**) molecular function; (**c**) biological process; and (**d**) cellular component. Analysis was performed using the PANTHER database program (http://www.pantherdb.org/). Black and gray bars indicate down-regulated and up-regulated proteins, respectively

Among the recent non-gel-based approaches, we applied isobaric TMT-based quantitative MS as this method is among the most popular quantitative proteomics as mentioned in the Introduction. The workflow of the quantitative proteomic study using six-plex TMT labeling following MS analysis is schematically illustrated in Fig. 2. In this scheme, the peptides digested from the sham, IR, and TALE groups were individually labeled with two different TMT tags to exclude technical variations in a single 2D-LC MS/MS experiment. The labeled peptides were equally mixed and subjected to identification and quantification via tandem MS. The proteins that showed reproducibility in the MS/MS results from two independent sets were considered, representing 641 non-redundant proteins (Fig. 4a). The information of MS data and proteins identified in two independent sets was listed in Additional file 1. The proteins differentially expressed between the sham-operation group and IR group were selected as IR-related proteins on the basis of significant log_2_ ratios > ±0.6 and the presence of two or more peptides. The sham-operated rats were used as controls. We identified 151 proteins and referred to the high stringency data sets as IR-related proteins (Additional file 2). In total, 62 proteins exhibited increased levels, whereas 89 proteins exhibited decreased levels. The IR-related proteins were further analyzed for Gene Ontology annotation and verification of the expression levels from proteome data.

### Gene ontology analysis of IR-related proteins

To determine the potential biological relevance of the IR-related proteins, we performed a Gene Ontology analysis using PANTHER to identify their molecular functions, biological processes, and cellular components (Fig. 4b-d, Additional file 3).

The molecular function analysis revealed that most proteins with reduced expression upon IR injury were related to catalytic activity (60.7%) (Fig. 4b). Concerning the biological processes, most proteins were assigned to the cellular process (37.0%) and metabolic process (33.3%) clusters (Fig. 4c). In particular, within metabolic processes, many proteins involved in glycolysis (Pgam1, Pgam2, Aldoa, Aldoc, Tpi1, Gpi, Pygm, Pgk1, and Pkm2) were significantly down-regulated in response to IR injury. Minor groups of proteins also clustered in biological regulation (7.4%), multicellular organismal process (6.2%), response to stimulus (4.9%), and developmental process (3.7%). The remaining 3 classes each had < 3% proteins assigned to these groups. The cellular component analysis revealed that most proteins were from cell compartments, such as cell part (66.7%), and organelle (19.0%) (Fig. 4d). Only a few proteins are released into the extracellular region.

In contrast, the results of the proteins increased in response to IR injury revealed an enrichment in protein binding (39.0%) and catalytic activity (33.9%) in the IR group (Fig. 4b). The cellular process (37.5%) was dominantly altered, and the other biological processes of these proteins included response to stimulus (15.3%), localization (12.5%), biological regulation (11.1%), and metabolic process (11.1%) in sequence order (Fig. 4c). The cellular components showed most proteins were assigned to extracellular region (48.5%), and cell part (30.3%) (Fig. 4d).

### Validation of TMT-based proteomic data

**Fig. 5:**
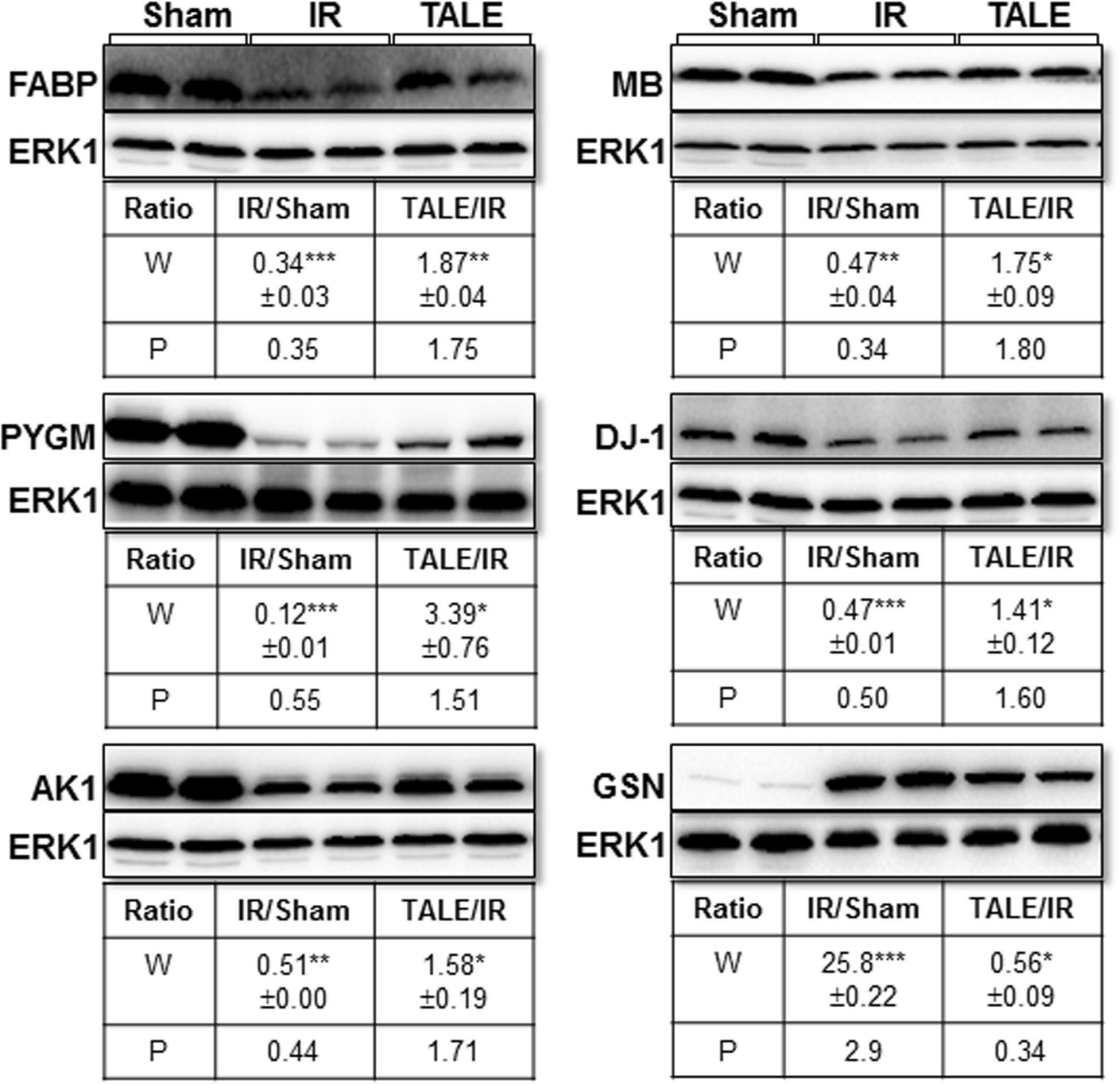
Validation of the expression levels of several proteome data analyses by a Western blot analysis. Representative Western blot results of FABP, PYGM, AK1, MB, DJ-1(Park7), and GSN are presented. The number indicates the average fold-changes of the quantitative Western blot results (W) from biological duplicates and quantitative proteome data (P). *P < 0.05, **P < 0.01, ***P < 0.001

Western blotting was performed to verify the expression level of several proteins identified by the TMT-based quantitative proteomics using new biological replicates of each group. We selected six IR-related proteins (differentially expressed proteins), including glycogen phosphorylase (PYGM), FABP, adenylate kinase isoenzyme 1 (AK1), myoglobin (MB), protein DJ-1 (Park7), and isoform 2 of gelsolin (GSN). Representative Western blot results are presented in Fig. 5. The expression levels of these proteins were analyzed using ImageJ software. FABP, PYGM, AK1, MB, and DJ-1 expression levels were significantly reduced in the IR group compared with those in the sham-operation group. In contrast, the GSN levels were significantly increased in the IR group. The direction of the expression level change in the Western blot results of these six proteins was consistent with the proteomic data; however, the magnitude of the change substantially differed for GSN. Also, the alteration of expression levels of these six proteins in Western results of TALE group corresponded well with that of the proteomic data. Thus, these results demonstrated the satisfactory quality of the proteomic experimental procedures and data.

### Effect of TALE on IR-related proteins

The effect of TALE on these IR-related proteins was further analyzed. The expression levels of proteins in the TALE group were quantified relative to those of the IR group to identify proteins affected by TALE upon IR injury. Total 57 of the IR-related proteins were influenced by TALE intake, representing 25 up-regulated proteins and 32 down-regulated proteins. TALE intake mainly exhibited a metabolic shift attenuating the loss of high-energy phosphate (ATP) and inhibiting the generation of reactive oxygen species (ROS). In addition, cytoskeleton impairment was inhibited by TALE intake.

### TALE intake efficiently generates ATP required for IR injury via a metabolic switch

**Fig. 6:**
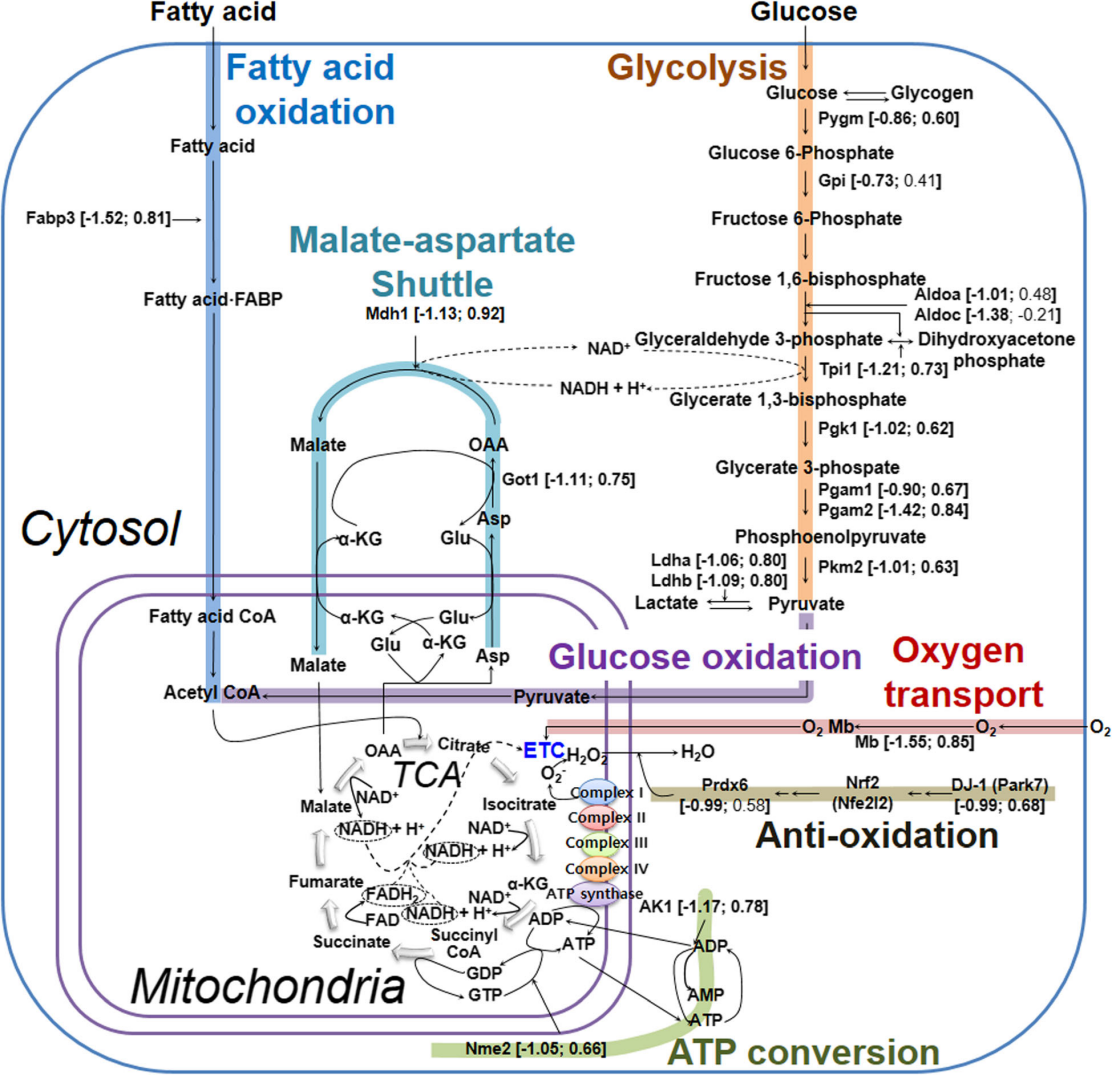
Metabolic adaptation for efficient ATP production in IR by TALE intake. Detailed information is described in the Results and Discussion. The numbers in parenthesis represent the quantitative values obtained by TMT-based quantitative proteomics and are presented as a fold-change change [log_2_ (IR versus sham); log_2_ (TALE versus IR)]. Abbreviations: FA, fatty acid,; α-KG, α-ketoglutarate,; Asp, aspartate; OAA, oxaloacetate; Glu, glutamic acid; ETC, electron transport chain; TCA,tricarboxylic acid cycle

Notably, the IR-related proteins are involved in ATP synthesis, namely, fatty acid oxidation, glycolysis, glucose oxidation, tricarboxylic acid (TCA) cycle, electron transport chain (ETC) and ATP conversion (Fig. 6). These results indicate a switch in the mode of ATP production during myocardial infarction (myocardial ischemia and reperfusion) and are consistent with the importance of these proteins for cardiac adaptation to IR injury [11]. Laboratory and clinical studies have suggested that cardiac metabolism is abnormal in patients with heart failure and is characterized by a diminished capacity to convert chemical energy into mechanical work due to mitochondrial imbalances [31]. Approximately 95% of ATP produced in the healthy heart is obtained from oxidative phosphorylation in mitochondria in the presence of oxygen, whereas approximately 5% of ATP is obtained from glycolysis in the cytoplasm in the absence of oxygen [32].

As illustrated in Fig. 6, acetyl CoA is the substrate for oxidative phosphorylation and is derived from the uptake of fatty acids into the cytosol mediated by fatty acid-binding protein, heart (Fabp3) and subsequent β-oxidation of fatty acids. Acetyl CoA is also derived from the hydrolysis of glycogen to generate glucose mediated by glycogen phosphorylase, muscle form (Pygm). Our proteome and Western results showed that these two enzymes (Fabp3 and Pygm) were increased by TALE intake. Then, pyruvate produced from glycolysis mediated by several increased enzymes [triosephosphate isomerase (Tpi1), phosphoglycerate kinase (Pgk1), phosphoglycerate mutase 1 and 2 (Pgam1 and 2), and pyruvate kinase (Pkm2)] and lactate oxidation mediated by enhaced lactate dehydrogenases (Ldha and Ldhb) in TALE intake could be generated to a high amount of acetyl CoA in mitochondria. The overproduced acetyl CoA is metabolized to generate carbon dioxide in the TCA cycle, during which NADH, FADH_2_, and GTP are highly produced. Also, NADH generated from glycolysis in the cytoplasm is transported into mitochondria through the malate-aspartate shuttle system, which is partially mediated by aspartate aminotransferase (Got1), and malate dehydrogenase (Mdh1) [33]. TALE intake up-regulated the expression of Got1 and Mdh1 on IR injury. Electrons on NADH and FADH_2_ are transferred through the ETC to oxygen to yield water at complex IV, where the oxygen supply is enhanced by myoglobin (Mb), ultimately leading to the synthesis of ATP by ATP synthase. In addition, ATP can be produced from GTP by nucleoside diphosphate kinase (Nme2) and ADP by adenylate kinase (Ak1). Proteome data showed these proteins (Mb, Nme2 and AK1) were up-regulated by TALE intake on IR injury. Moreover, the expression levels of two proteins (Mb and Ak1) verified by Western blots were consistent with those of proteomic data. Thus, these results suggest that TALE intake enhances ATP production. In fact, sharply reduced ATP production is particularly severe for cardiac tissue viability and function. In ischemia, most ATP is produced through anaerobic glycolysis due to an insufficient oxygen supply. However, in reperfusion, ATP production through the oxidation of fatty acid and glucose is recovered based on the oxygen supply [32, 34]. Therefore, our results reveal that the key to improving cardiac function after IR is to restore robust ATP production through oxidative phosphorylation via efficient ETC and ATP conversion.

Furthermore, the generation of superoxide (O_2_^−^), which is a subset of ROS, is greatly increased at the ETC (complex I and III in particular). Superoxide can damage enzymes involved in ATP generation (Fig. 6). The deleterious effects of superoxide can be eliminated by antioxidant systems. For example, DJ-1 (Park7) releases nrf2 from its inhibitor Keap1 [35]. Free nrf2 translocates to the nucleus and upregulates the transcription of target genes, including peroxiredoxin 6 (Prdx 6) [36]. Subsequently, the peroxiredoxin proteins transform hydrogen peroxide generated from superoxide into innocuous water. TALE intake up-regulated the expression of DJ-1 and peroxiredoxin 6, which could redcue the ROS level in mitochondria. Thus, TALE contributes to maintaining the normal functions of enzymes involved in ATP generation. This hypothesis was also demonstrated in our previous study. Specifically, wheat extract attenuated ROS production and subsequent apoptosis in human blastoma cells [17], indicating that it acts as an antioxidant by removing ROS formed during IR [37] and inhibits downstream apoptotic cascades by preventing malondialdehyde (MDA) production from lipid peroxidation due to ROS [19, 38].

Taken together, TALE intake contributes to maintaining high ATP levels in IR through the upregulation of enzyme expression and the maintenance of normal enzymatic functions.

### TALE reduces cytoskeleton impairment

**Fig. 7:**
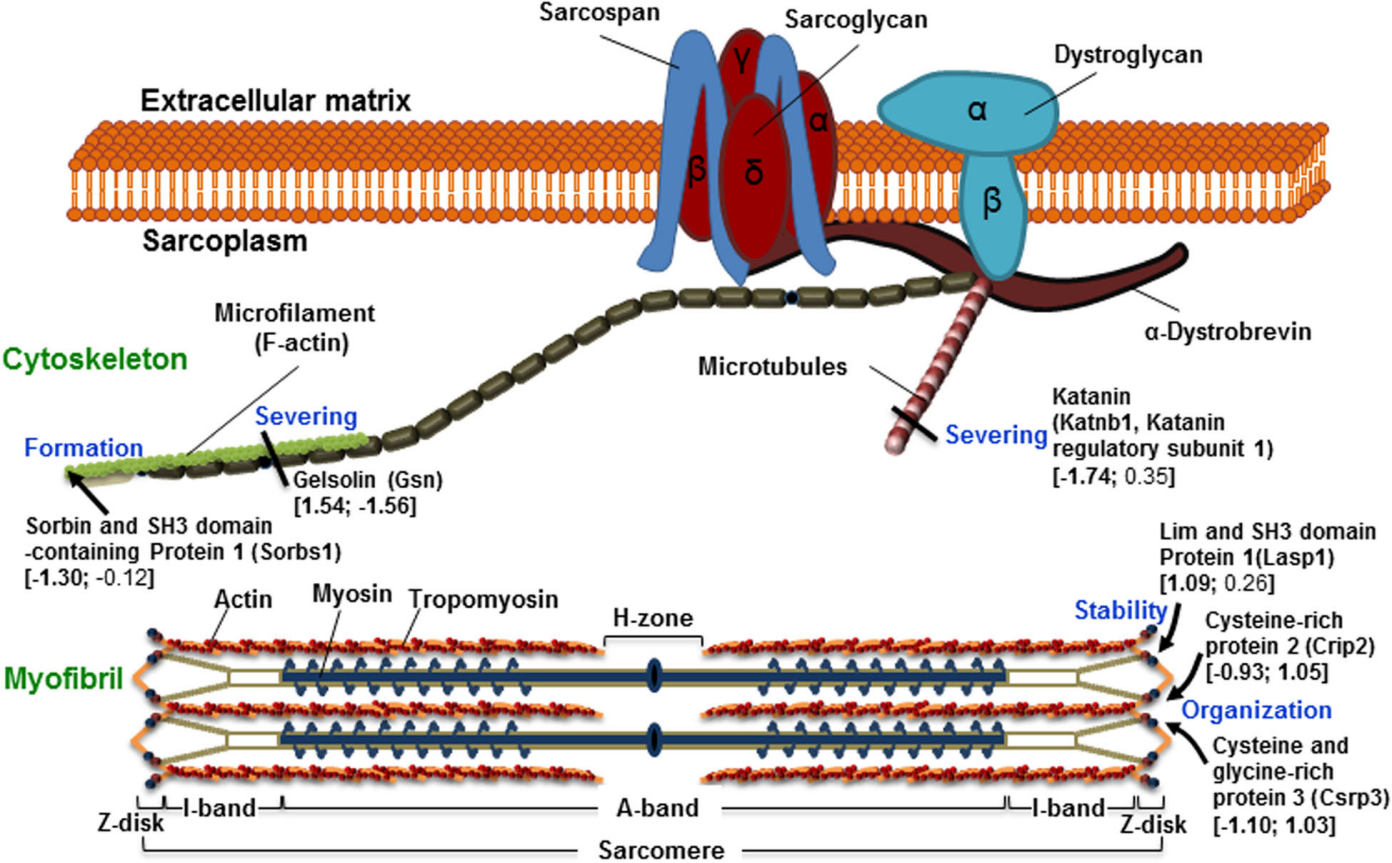
Cytoskeleton disruption in IR is inhibited by TALE intake. The image of the cytoskeleton structure and proteins is modified from [41]. The number shown in parenthesis indicates the quantitative values were obtained by TMT-based quantitative proteomics and is presented as a fold-change [log_2_ (IR versus sham); log_2_ (TALE versus IR)]

The cardiac cytoskeleton is divided into contractile and cytoskeletal components [39]. Myofibrils are the major contractile components responsible for the conversion of chemical energy (ATP) into mechanical energy. Myofibrils consist of sarcomere repeating units that can be subdivided into contractile proteins, such as actin and myosin, and structural scaffolding proteins, such as Fhl2 (four and one-half LIM domains), Crip2 (Cysteine-rich protein 2), Csrp3 (Cysteine and glycine-rich protein 3), and Lasp1 (LIM and SH3 domain protein 1), which are located in the Z-disk in addition to other regions [40] (Fig. 7). Some proteins are involved in Z-disc stability, such as Lasp1 [42]; Z-disc organization, such as Crip2; and sarcomere assembly, such as Csrp3 [40]. The Fhl2, Crip2, and Csrp3 were significantly decreased by IR injury. In contrast, the Crip2 and Csrp3 were highly increased by TALE intake on IR injury.

The cytoskeletal components include microfilaments, such as actin, and microtubules composed of tubulin. Microfilaments and microtubules undergo polymerization and depolymerization to adjust to environmental stresses. Specifically, gelsolin and katanin sever microfilaments and microtubules, respectively [43, 44], and Sorb1 (Sorbin and SH3 domain-containing protein 1) stimulates microfilament polymerization [45]. The katanin and Sorb1 were significantly decreased, while gelsolin was increased by IR injury (Fig. 7). However, TALE intake significantly reduced the synthesis of gelsolin, as shown in proteomic and Western results. Therefore, TALE intake contributes to cardiomyocyte survival in ischemic heart disease by stabilizing the myofibril structure and adjusting the microfilament and microtubule levels in cardiomyocytes because the cardiac cytoskeleton becomes damaged, especially during reperfusion in myocardial infarction [46].

## Conclusions

The results of this study obtained using TMT-based quantitative proteomics suggest that cardiac IR induces significant alterations in the moderators of cell metabolism and host defense in the heart. In particular, these proteins perform important functions in metabolic pathways and several cellular enzyme cascades important for the response to stress and the cytoskeleton. Furthermore, TALE intake affects metabolic adaptation for the efficient production of high-energy phosphate (ATP) in conjunction with reduced generation of reactive oxygen species (ROS) and cytoskeleton disruption, thereby reducing cardiac IR injury. Therefore, these findings provide a more complete picture of the biological roles of TALE in both IR injury and other aspects of cellular biology.

## Supplementary information

**Additional file 1.** List of MS data and proteins indentified in two independent sets by TMT-based quantitative proteomics.

**Additional file 2.** List of IR-related proteins indentified based on the high stringency criteria. The fold-change (log_2_ scale) is calculated by averaging the two ratio values of IR versus sham or TALE versus IR. No. of peptides indicates the number of independent spectra used for the quantification.

**Additional file 3.** PANTHER analysis results of the differentially expressed proteins in IR injury.

## Data Availability

The datasets supporting the conclusions of this article are included within the article and its additional files.

## Abbreviations

2-DE: Two-dimensional gel electrophoresis
2D-LC: Two-dimensional liquid chromatography
AAR: Areas at risk
CHAPS: 3-[(3-cholamidopropyl) dimethylammonio]-1-propanesulfonate
CID: Collision induced dissociation
DF: Dietary fiber
DTT: Dithiothreitol
ETC: Electron transport chain
FDR: False discovery rate
HCD: High energy collision dissociation
IA: Infarct area
IPG: Immobilized pH gradient
IR: Ischemia-reperfusion
IS: Infarct size
iTRAQ: Isobaric tags for relative and absolute quantitation
JNK: c-Jun N-terminal kinase
LAD: Left anterior descending coronary artery
LC: Liquid chromatography
LSD: Least significant difference
LTQ: Linear trap quadrupole
LV: Left ventricle
MAP: Mitogen-activated protein
MS: Mass spectrometry
MS/MS: Tandem mass spectrometry
PANTHER: Protein analysis through evolutionary relationships
ROS: Reactive oxygen species
RS: Risk size
SCX: Strong cation exchange
SD: Sprague Dawley
SDS-PAGE: Sodium dodecyl sulfate polyacrylamide gel electrophoresis
TALE: *Triticum aestivum* L. extract
TCA: Tricarboxylic acid
TMT: Tandem mass tag
TTC: 2,3,5-triphenyltetrazolium chloride

## References

[1] Shimokawa H, Yasuda S (2008). Myocardial ischemia: current concepts and future perspectives. J Cardiol.

[2] Lopez AD, Mathers CD, Ezzati M, Jamison DT, Murray CJ (2006). Global and regional burden of disease and risk factors, 2001: systematic analysis of population health data. Lancet.

[3] Bao N, Ushikoshi H, Kobayashi H, Yasuda S, Kawamura I, Iwasa M (2009). Simvastatin reduces myocardial infarct size via increased nitric oxide production in normocholesterolemic rabbits. J Cardiol.

[4] Tokuyama T, Sakuma T, Motoda C, Kawase T, Takeda R, Mito S (2009). Intravenous administration of adenosine triphosphate disodium during primary percutaneous coronary intervention attenuates the transient rapid improvement of myocardial wall motion, not myocardial stunning, shortly after recanalization in acute anterior myocardial infarction. J Cardiol.

[5] Zhao ZQ, Vinten-Johansen J (2006). Postconditioning: reduction of reperfusion-induced injury. Cardiovasc Res.

[6] Heusch G, Libby P, Gersh B, Yellon D, Bohm M, Lopaschuk G (2014). Cardiovascular remodelling in coronary artery disease and heart failure. Lancet.

[7] Sakai J, Ishikawa H, Satoh H, Yamamoto S, Kojima S, Kanaoka M (2007). Two-dimensional differential gel electrophoresis of rat heart proteins in ischemia and ischemia-reperfusion. Methods Mol Biol.

[8] Yue QX, Xie FB, Song XY, Wu WY, Jiang BH, Guan SH (2012). Proteomic studies on protective effects of salvianolic acids, notoginsengnosides and combination of salvianolic acids and notoginsengnosides against cardiac ischemic-reperfusion injury. J Ethnopharmacol.

[9] Fert-Bober J, Basran RS, Sawicka J, Sawicki G (2008). Effect of duration of ischemia on myocardial proteome in ischemia/reperfusion injury. Proteomics.

[10] Cadete VJ, Lin HB, Sawicka J, Wozniak M, Sawicki G (2012). Proteomic analysis of right and left cardiac ventricles under aerobic conditions and after ischemia/reperfusion. Proteomics.

[11] Li X, Arslan F, Ren Y, Adav SS, Poh KK, Sorokin V (2012). Metabolic adaptation to a disruption in oxygen supply during myocardial ischemia and reperfusion is underpinned by temporal and quantitative changes in the cardiac proteome. J Proteome Res.

[12] Warren CM, Geenen DL, Helseth DL, Xu H, Solaro RJ (2010). Sub-proteomic fractionation, iTRAQ, and OFFGEL-LC-MS/MS approaches to cardiac proteomics. J Proteome.

[13] Merali Z, Gao MM, Bowes T, Chen J, Evans K, Kassner A (2014). Neuroproteome changes after ischemia/reperfusion injury and tissue plasminogen activator administration in rats: a quantitative iTRAQ proteomics study. PLoS One.

[14] Threapleton DE, Greenwood DC, Evans CE, Cleghorn CL, Nykjaer C, Woodhead C (2013). Dietary fibre intake and risk of cardiovascular disease: systematic review and meta-analysis. BMJ.

[15] Bazzano LA, He J, Ogden LG, Loria CM, Whelton PK, National H (2003). Dietary fiber intake and reduced risk of coronary heart disease in US men and women: the National Health and nutrition examination survey I epidemiologic follow-up study. Arch Intern Med.

[16] Han HS, Jang JH, Jang JH, Choi JS, Kim YJ, Lee C (2010). Water extract of Triticum aestivum L. and its components demonstrate protective effect in a model of vascular dementia. J Med Food.

[17] Jang JH, Kim CY, Lim SH, Yang CH, Song KS, Han HS (2010). Neuroprotective effects of Triticum aestivum L. against beta-amyloid-induced cell death and memory impairments. Phytother Res.

[18] Lim SH, Kim MY, Lee J (2014). Apple pectin, a dietary fiber, ameliorates myocardial injury by inhibiting apoptosis in a rat model of ischemia/reperfusion. Nutr Res Pract.

[19] Lim SH, Kim Y, Yun KN, Kim JY, Jang JH, Han MJ (2016). Plant-based foods containing cell wall polysaccharides rich in specific active monosaccharides protect against myocardial injury in rat myocardial infarction models. Sci Rep.

[20] Lim SH, Song KS, Lee J (2010). Butyrate and propionate, short chain fatty acids, attenuate myocardial damages by inhibition of apoptosis in a rat model of ischemia-reperfusion. J Korean Soc Appl Biol Chem.

[21] Lim SH, Lee J (2012). Methanol extract of Cassia mimosoides var. nomame attenuates myocardial injury by inhibition of apoptosis in a rat model of ischemia-reperfusion. Prev Nutr Food Sci.

[22] Han MJ, Lee SY, Hong SH (2012). Comparative analysis of envelope proteomes in Escherichia coli B and K-12 strains. J Microbiol Biotechnol.

[23] Lee J, Jang YS, Han MJ, Kim JY, Lee SY. Deciphering Clostridium tyrobutyricum metabolism based on the whole-genome sequence and proteome analyses. mBio. 2016;7:1-12. 10.1128/mBio.00743-16 PMC4916380 27302759

[24] Washburn MP, Wolters D, Yates JR (2001). Large-scale analysis of the yeast proteome by multidimensional protein identification technology. Nat Biotechnol.

[25] Carvalho PC, Xu T, Han X, Cociorva D, Barbosa VC, Yates JR (2009). YADA: a tool for taking the most out of high-resolution spectra. Bioinformatics.

[26] Li Z, Adams RM, Chourey K, Hurst GB, Hettich RL, Pan C (2012). Systematic comparison of label-free, metabolic labeling, and isobaric chemical labeling for quantitative proteomics on LTQ Orbitrap Velos. J Proteome Res.

[27] Tabb DL, McDonald WH, Yates JR (2002). DTASelect and contrast: tools for assembling and comparing protein identifications from shotgun proteomics. J Proteome Res.

[28] Raso C, Cosentino C, Gaspari M, Malara N, Han X, McClatchy D (2012). Characterization of breast cancer interstitial fluids by TmT labeling, LTQ-Orbitrap Velos mass spectrometry, and pathway analysis. J Proteome Res.

[29] Van Eyk JE (2011). Overview: the maturing of proteomics in cardiovascular research. Circ Res.

[30] Rentrop KP, Feit F (2015). Reperfusion therapy for acute myocardial infarction: concepts and controversies from inception to acceptance. Am Heart J.

[31] Walters AM, Porter GA, Brookes PS (2012). Mitochondria as a drug target in ischemic heart disease and cardiomyopathy. Circ Res.

[32] Lopaschuk GD (2017). Metabolic modulators in heart disease: past, present, and future. Can J Cardiol.

[33] Fu C, Wu C, Liu T, Ago T, Zhai P, Sadoshima J (2009). Elucidation of thioredoxin target protein networks in mouse. Mol Cell Proteomics.

[34] Solaini G, Harris DA (2005). Biochemical dysfunction in heart mitochondria exposed to ischaemia and reperfusion. Biochem J.

[35] Raninga PV, Trapani GD, Tonissen KF (2014). Cross talk between two antioxidant systems, Thioredoxin and DJ-1: consequences for Cancer. Oncoscience.

[36] Murphy KE, Park JJ. Can co-activation of Nrf2 and Neurotrophic signaling pathway slow Alzheimer's disease? Int J Mol Sci. 2017;18:1-28. 10.3390/ijms18061168 PMC5485992 28561773

[37] Weismann D, Hartvigsen K, Lauer N, Bennett KL, Scholl HP, Charbel Issa P (2011). Complement factor H binds malondialdehyde epitopes and protects from oxidative stress. Nature.

[38] Ebert AD, Kodo K, Liang P, Wu H, Huber BC, Riegler J (2014). Characterization of the molecular mechanisms underlying increased ischemic damage in the aldehyde dehydrogenase 2 genetic polymorphism using a human induced pluripotent stem cell model system. Sci Transl Med.

[39] Sequeira V, Nijenkamp LL, Regan JA, van der Velden J (1838). The physiological role of cardiac cytoskeleton and its alterations in heart failure. Biochim Biophys Acta.

[40] Li A, Ponten F, dos Remedios CG (2012). The interactome of LIM domain proteins: the contributions of LIM domain proteins to heart failure and heart development. Proteomics.

[41] Rahimov F, Kunkel LM (2013). The cell biology of disease: cellular and molecular mechanisms underlying muscular dystrophy. J Cell Biol.

[42] Fernandes I, Schock F (2014). The nebulin repeat protein Lasp regulates I-band architecture and filament spacing in myofibrils. J Cell Biol.

[43] Nag S, Larsson M, Robinson RC, Burtnick LD (2013). Gelsolin: the tail of a molecular gymnast. Cytoskeleton (Hoboken).

[44] Ghosh DK, Dasgupta D, Guha A (2012). Models, regulations, and functions of microtubule severing by Katanin. ISRN Mol Biol.

[45] Kioka N, Ueda K, Amachi T (2002). Vinexin, CAP/ponsin, ArgBP2: a novel adaptor protein family regulating cytoskeletal organization and signal transduction. Cell Struct Funct.

[46] Perricone AJ, Vander Heide RS (2014). Novel therapeutic strategies for ischemic heart disease. Pharmacol Res.

